# Reassessment of fluctuating dental asymmetry in Down syndrome

**DOI:** 10.1038/s41598-017-16798-0

**Published:** 2017-11-30

**Authors:** Marcos Matabuena Rodríguez, Pedro Diz Dios, Carmen Cadarso-Suárez, Márcio Diniz-Freitas, Mercedes Outumuro Rial, Maria Teresa Abeleira Pazos, Jacobo Limeres Posse

**Affiliations:** 1 Unit of Biostatistics, Department of Statistics and Operations Research, School of Medicine, University of Santiago de Compostela (USC), Santiago de Compostela, Spain; 2 Medical-Surgical Dentistry Research Group (OMEQUI), Health Research Institute of Santiago de Compostela (IDIS), University of Santiago de Compostela (USC), Santiago de Compostela, Spain; 3 Rede Galega INBIOEST, Santiago de Compostela, Spain

**Keywords:** Developmental biology, Oral anatomy

## Abstract

Fluctuating dental asymmetry (FDA) is a tool to measure developmental stability that could be increased in gonosomal aneuploidies. The aim of this study was to quantify FDA in individuals with Down syndrome (DS). The study group comprised 40 individuals with DS, and a control group matched for age and sex was created. The target teeth were the maxillary central incisors (11,21), maxillary lateral incisors (12,22), maxillary canines (13,23), and maxillary first molars (16,26). Dental morphometric variables measured on CBCT images included tooth length, crown height, root length, mesio-distal diameter, crown-to-root ratio, vestibular-palatine diameter, mid mesio-distal diameter, mid buccal-palatal diameter, maximum buccal-palatal diameter, and cervical circumference. The FA2 fluctuating asymmetry index (Palmer and Strobeck, 1986) was applied. Some discrepancies in crown-to-root ratios and root length asymmetry were significantly lower in the DS individuals than in controls. Combining the crown-to-root ratio of tooth 11 versus 21, tooth 12 versus 22, and tooth 13 versus 23, we developed a predictive model with a discriminatory power between DS and controls of 0.983. Some dental morphometric variables may actually be more stable in DS individuals than in the general population. This offers a new perspective on the relationship between canalization, fluctuating asymmetry, and aneuploidy.

## Introduction

The concept of *canalization* refers to a phenomenon whereby the development of phenotypic traits is buffered against environmental influences, so that such traits produce a highly predictable genetically determined endpoint^1^. The canalization capacity of an organism is called *developmental stability* ^2^ and its measurement is based on small variations of antimeric traits at random with respect to side (right-left). This biological asymmetry of morphological traits is called *fluctuating asymmetry* ^3^.

It has been suggested that canalization is reduced, and fluctuating asymmetry thus augmented, in disorders of developmental origin, and is detectable in most if not all of the gonosomal aneuploidies^4,5^. The most common live-born human aneuploidy is trisomy 21, which causes Down syndrome (DS). The term *amplified developmental instability* was coined around 50 years ago to describe the generalized genetic imbalance that trisomy 21 causes in developmental homeostasis^6^. In DS, increased fluctuating asymmetry has been reported in skeletal anomalies^7^, dermatoglyphics^8^, facial dysmorphology^9^, and palatal dimensions^10^.

Application of the concept of fluctuating asymmetry to teeth has enabled small, randomly distributed morphometric differences to be identified between the teeth of contralateral arches; this is called *fluctuating dental asymmetry* ^11^. Few details of the genetic and environmental factors implicated in fluctuating dental asymmetry are yet known^12^, with the exception of chromosomal abnormalities and some single gene substitutions^13,14^. Articles published in the 1970s and 80s showed that individuals with DS had significantly greater tooth crown asymmetry than controls^13,15,16^. One of the drawbacks of those studies was that only the crown dimensions were evaluated. It has been stated that the study of fluctuating asymmetry requires the selection of traits with a low vulnerability to wear, as this would otherwise complicate the interpretation of asymmetry variation^17^, and tooth crown wear is paradoxically particularly common and severe in DS due to attrition and erosion^18^. A further limitation common to those studies was that other variables that could affect tooth morphometrics and asymmetry, such as sexual dimorphism^19^ or age^20^, were not taken into account.

In 2014, we published an article in which we analyzed tooth dimensions not previously studied in individuals with DS—such as root length and cervical circumference—using cone beam computed tomography (CBCT) images^21^. We found significant differences in crown height and crown-to-root ratio between the maxillary right and left canines, and in maximum buccal-palatal diameter between the maxillary right and left first molars^21,22^. In that study, the results were analyzed using additive mixed models^23^, which enabled us to include the smooth effect of age, the fixed effects of sex and teeth, and the random effect of patient. The main drawback of that study was that asymmetry of the dental morphometric variables was measured as the absolute value of the difference between right and left, while the most useful descriptor of fluctuating asymmetry is variance^3,24^. The aim of the present study has been to reassess fluctuant dental asymmetry in a series of individuals with DS, evaluated objectively without taking into account the absolute size of the teeth and, therefore, without the effect of scale.

## Material and Methods

**Figure 1:**
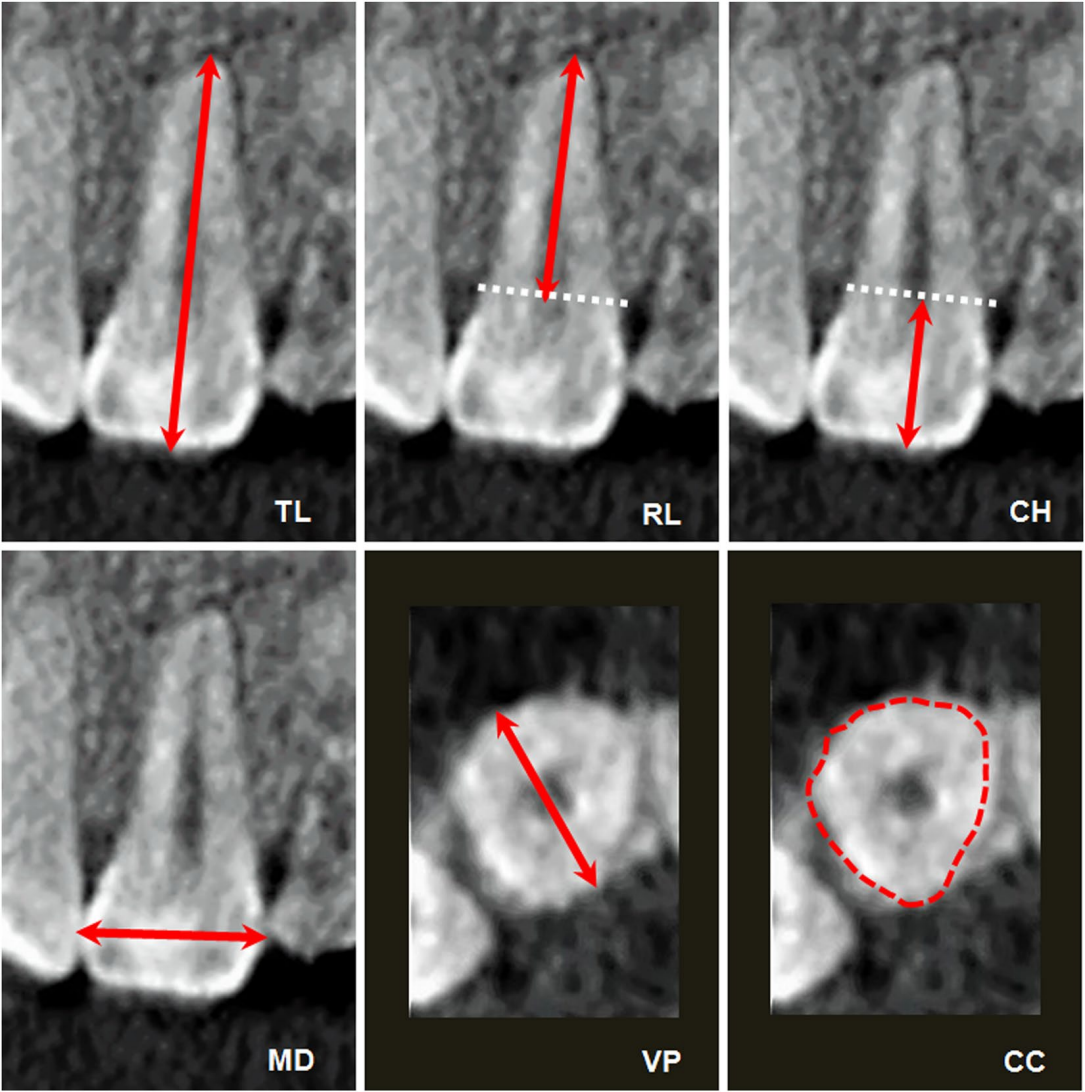
Measurement of some relevant dental dimensions: overall tooth length (TL), crown height (CH), root length (RL), mesio-distal diameter (MD), vestibular-palatine diameter (VP), and cervical circumference (for further details see reference Abeleira *et al*.^21^).

The characteristics of the study group and the methodology used to obtain the CBCT image are described in detail in our previous article^21^. Briefly, the study group was formed of 40 white individuals with DS (25 males and 15 females; mean age, 18.8 ± 7.3 years [range, 9–43 years]). The control group comprised 40 healthy, age- and sex-matched individuals without DS (25 males and 15 females; mean age, 19.5 ± 7.2 years [range, 10–43 years]). The CBCT images were obtained using an I-CAT**®** scanner (Imaging Sciences International, Hatfield, PA, USA), were reconstructed with I-CAT VISION^**®**^ software (Imaging Sciences International), and were exported using the DICOM (Digital Imaging Communication in Medicine) format to a MacBook 27 personal computer (Mac OsX 10.6, Apple, Inc., Cupertino, USA). Measurements were performed using the open-source OsiriX medical image processing software (Pixmeo, Geneva, Switzerland; www.osirixviewer.com). For the analysis of tooth morphometry, the CBCT images were oriented using multiplanar reconstruction and a modification of the method described by Sherrard *et al*.^25^ was applied. The target teeth of the study were the maxillary central incisors, the maxillary lateral incisors, the maxillary canines, and the palatal root of the maxillary first molars. Overall tooth length, crown height, root length, and mesio-distal diameter were measured in the coronal plane. The crown-to-root ratio was defined as the ratio of the crown height to the root length. Vestibular-palatine diameter, mid mesio-distal diameter, mid buccal-palatal diameter, maximum buccal-palatal diameter, and cervical circumference were measured in the axial plane (Fig. 1). The interobserver and intraobserver reliability of this measurement system has been demonstrated previously^21^.

All the statistical analyses were carried out with the statistical software R, version 2.12.0. (R Development Core Team, Vienna, Austria), using the following packages: “fBasics” to calculate the basic statistics of each of the variables analyzed; and “mgcv” to fit additive mixed models to determine dimensional symmetry between the central incisors, between the lateral incisors, between the canines, and between the first molars. Additive mixed models are extensions of linear mixed models and enable us to include random effects in addition to the usual fixed effects^26^. In this study, we considered the following additive mixed models, which included the smooth effect of age, the fixed effects of gender and teeth, and the random effect of patient: [Tooth measurement = β_0_ + f (Age) + Gender + Teeth + random (Patient) + ε], where f (.) refers to unspecified smooth functions, producing separate effects of age in each group.

Hypothesis testing was performed using a variety of methods (t test, Wilcoxon, Kruskal-Wallis) to determine whether significant differences in tooth dimensions existed between the study group (DS) and the control group.

At the individual level, we used the FA2 fluctuating asymmetry index described by Palmer and Strobeck [3,27]). In this index, the |Right - Left| difference is divided by the mean, given as (R + L)/2. This fluctuating asymmetry index corrects for trait size effects by expressing deviations from symmetry as a proportion of trait size^17^. The FA2 results underwent further hypothesis testing, mainly using the Kruskal-Wallis test as some data did not have a normal distribution, to determine whether significant differences in the degree of dental asymmetry were present between the DS group and the control group.

A generalized additive model was developed to evaluate the discriminatory power of the dental morphometric variables, processed using the FA2 index, to classify a given individual as DS or non-syndromic control.

These radiological studies were performed in accordance with the radiation protection principles of *As Low As Reasonably Achievable (ALARA)* and following the guidelines of the SEDENTEXCT Guideline Development Panel. Radiation Protection No. 172: Cone Beam CT for Dental and Maxillofacial Radiology. Evidence Based Guidelines 2012 (www.sedentexct.eu).”

### Ethical approval

The study was approved by the Institutional Review Board of the University of Santiago de Compostela (USC), Spain.

### Informed consent

All the images used in this study belonged to the historical archive of the Radiology Unit of the Faculty of Medicine and Dentistry of the University of Santiago de Compostela in Spain. No specific informed consent was required as all participants or, as appropriate, their legal representatives had signed an informed consent to authorize the use of images for teaching or research purposes.

### Data availability

The datasets generated during and/or analysed during the current study are available from the corresponding author on reasonable request.

## Results

The absolute values of the dental morphometric variables evaluated in the DS group and the control group are shown in Table 1. The Kruskal-Wallis test revealed statistically significant differences between individuals with DS and controls in 69 of the 74 dental morphometric variables evaluated (Table 2). With the exception of the crown-to-root ratio of teeth 11, 21, and 16, the root length of tooth 22, and the crown height of tooth 26, all values were significantly lower in the individuals with DS.

**Table 1:** Dental morphometric variables in individuals with Down syndrome and controls (in millimeters).

Tooth	Dimension	Down Syndrome					Controls				
		Mean	Median	Standard deviation	Minimum	Maximum	Mean	Median	Standard deviation	Minimum	Maximum
Maxillary right central incisor (11)	Overall tooth length	1.911	1.916	0.232	1.391	2.547	1.952	2.149	0.586	0.640	2.563
	Crown height	0.838	0.816	0.134	0.474	1.153	1.111	1.088	0.221	0.704	1.787
	Root length	1.072	1.087	0.196	0.558	1.443	1.120	1.127	0.238	0.663	1.471
	Mesio-distal diameter	0.776	0.755	0.081	0.591	0.953	0.830	0.834	0.074	0.702	0.955
	Vestibular-palatine diameter	0.676	0.668	0.066	0.508	0.892	0.741	0.737	0.107	0.358	0.879
	Crown-to-root ratio	0.461	0.454	0.093	0.248	0.844	1.223	0.996	0.709	0.541	2.662
	Cervical circumference	2.303	2.305	0.182	1.993	2.824	2.489	2.491	0.161	2.039	2.818
Maxillary right lateral incisor (12)	Overall tooth length	1.521	1.521	0.305	0.882	2.294	1.916	1.910	0.303	0.932	2.644
	Crown height	0.775	0.620	0.973	0.103	6.543	0.809	0.798	0.154	0.581	1.370
	Root length	0.896	0.904	0.273	0.228	1.525	1.138	1.171	0.270	0.532	1.591
	Mesio-distal diameter	0.651	0.580	0.323	0.327	1.929	0.656	0.652	0.085	0.462	0.930
	Vestibular-palatine diameter	0.584	0.581	0.070	0.449	0.731	0.656	0.639	0.074	0.452	0.795
	Crown-to-root ratio	0.402	0.385	0.092	0.286	0.741	0.757	0.651	0.350	0.345	1.730
	Cervical circumference	1.849	1.914	0.408	0.246	2.586	2.106	2.087	0.152	1.723	2.427
Maxillary right canine (13)	Overall tooth length	1.628	1.582	0.316	0.696	2.122	2.073	1.981	0.387	1.383	2.948
	Crown height	0.629	0.625	0.108	0.357	0.887	0.846	0.809	0.214	0.564	1.366
	Root length	0.982	1.000	0.275	0.287	1.582	1.228	1.170	0.386	0.577	2.004
	Mesio-distal diameter	0.684	0.675	0.092	0.467	1.039	0.762	0.749	0.074	0.673	0.941
	Vestibular-palatine diameter	0.675	0.685	0.060	0.519	0.867	0.800	0.784	0.055	0.706	0.935
	Crown-to-root ratio	0.433	0.391	0.182	0.258	1.396	0.771	0.633	0.419	0.353	1.811
	Cervical circumference	2.189	2.208	0.323	0.657	2.786	2.485	2.550	0.181	2.061	3.041
Maxillary right first molar (16)	Overall tooth length	18.570	18.660	1.504	14.562	22.191	21.093	20.979	1.921	11.422	23.761
	Crown height	6.007	6.081	0.571	5.081	7.440	7.071	7.265	0.645	5.110	8.253
	Root length	12.499	12.383	1.572	8.132	15.911	14.267	14.284	0.830	13.457	15.955
	Mid mesio-distal diameter	9.682	9.673	0.398	9.062	10.782	9.987	9.897	0.654	8.830	11.420
	Mid buccal-palatal diameter	9.438	9.415	0.608	8.365	10.773	10.802	10.482	1.076	8.440	13.721
	Maximum buccal-palatal diameter	9.811	9.745	0.514	8.950	10.964	11.474	11.398	0.883	9.169	13.761
	Crown-to-root ratio	0.483	0.450	0.087	0.378	0.750	0.493	0.543	0.051	0.391	0.601
	Cervical circumference	29.488	29.278	1.450	26.861	32.516	32.798	32.855	1.806	29.192	35.620
Maxillary left central incisor (21)	Overall tooth length	1.885	1.884	0.254	1.333	2.563	1.947	2.194	0.589	0.975	2.601
	Crown height	0.861	0.859	0.135	0.490	1.209	1.115	1.097	0.152	0.865	1.395
	Root length	1.010	1.015	0.242	0.602	1.866	1.124	1.142	0.240	0.798	1.501
	Mesio-distal diameter	0.789	0.781	0.083	0.647	1.082	0.832	0.811	0.062	0.740	0.986
	Vestibular-palatine diameter	0.678	0.672	0.062	0.553	0.891	0.762	0.741	0.071	0.683	0.914
	Crown-to-root ratio	0.458	0.455	0.063	0.325	0.581	1.044	0.914	0.465	0.208	2.009
	Cervical circumference	2.328	2.318	0.184	1.974	2.617	2.457	2.514	0.263	2.009	2.939
Maxillary left lateral incisor (22)	Overall tooth length	1.622	1.658	0.268	1.113	2.086	1.939	1.966	0.367	1.239	2.695
	Crown height	0.643	0.637	0.180	0.370	1.075	0.822	0.775	0.143	0.562	1.075
	Root length	0.988	0.973	0.233	0.528	1.437	1.120	1.214	0.340	0.539	1.820
	Mesio-distal diameter	0.582	0.554	0.107	0.420	0.932	0.665	0.663	0.119	0.436	0.883
	Vestibular-palatine diameter	0.587	0.566	0.079	0.470	0.826	0.668	0.664	0.091	0.382	0.826
	Crown-to-root ratio	0.391	0.392	0.079	0.240	0.613	0.788	0.665	0.302	0.371	1.708
	Cervical circumference	1.871	1.872	0.346	0.265	2.801	2.204	2.199	0.314	1.612	2.896
Maxillary left canine (23)	Overall tooth length	1.649	1.662	0.344	0.814	2.345	2.080	1.913	0.483	1.140	3.377
	Crown height	0.624	0.616	0.100	0.385	0.842	0.785	0.791	0.105	0.536	0.997
	Root length	1.015	1.007	0.303	0.246	1.635	1.293	1.202	0.428	0.566	2.455
	Mesio-distal diameter	0.655	0.664	0.059	0.512	0.794	0.768	0.748	0.069	0.618	0.895
	Vestibular-palatine diameter	0.673	0.673	0.060	0.509	0.817	0.800	0.802	0.060	0.667	0.914
	Crown-to-root ratio	0.410	0.385	0.118	0.207	0.908	0.662	0.622	0.233	0.339	1.494
	Cervical circumference	2.166	2.161	0.174	1.837	2.798	2.610	2.625	0.212	2.200	3.151
Maxillary left first molar (26)	Overall tooth length	18.751	18.585	1.539	15.010	21.680	21.380	21.074	0.982	19.891	24.110
	Crown height	6.125	6.195	0.734	4.011	7.321	7.085	6.996	0.522	6.112	8.393
	Root length	12.627	12.955	1.464	7.690	15.364	14.306	14.085	0.693	13.070	15.894
	Mid mesio-distal diameter	9.748	9.555	0.671	8.783	12.246	10.305	10.008	0.786	8.946	12.320
	Mid buccal-palatal diameter	9.598	9.635	0.664	8.006	10.908	10.585	10.512	1.048	8.627	13.950
	Maximum buccal-palatal diameter	10.015	10.015	0.517	8.731	10.890	11.556	11.465	0.962	10.177	14.374
	Crown-to-root ratio	0.486	0.461	0.109	0.312	0.950	0.491	0.497	0.037	0.418	0.564
	Cervical circumference	29.593	29.475	1.497	26.752	32.372	32.241	32.778	1.548	29.245	35.319

**Table 2:** Statistical significance of the differences in dental morphometric variables between individuals with Down syndrome the control group.

Tooth	Dimension	T test	Wilcoxon test	Kruskal-Wallis test
Maxillary right central incisor (11)	Overall tooth length	0	0	0
	Crown height	0	0	0
	Root length	0.001	0	0
	Mesio-distal diameter	0.695	0.013	0.004
	Vestibular-palatine diameter	0	0	0
	Crown-to-root ratio	0.346	0.439	0.333
	Cervical circumference	0.004	0.003	0.003
Maxillary right lateral incisor (12)	Overall tooth length	0	0	0
	Crown height	0	0	0
	Root length	0	0	0
	Mesio-distal diameter	0	0	0
	Vestibular-palatine diameter	0.831	0	0
	Crown-to-root ratio	0	0	0.004
	Cervical circumference	0.918	0.001	0
Maxillary right canine (13)	Overall tooth length	0	0	0
	Crown height	0	0	0
	Root length	0	0	0
	Mesio-distal diameter	0	0	0
	Vestibular-palatine diameter	0	0	0
	Crown-to-root ratio	0.002	0.005	0.001
	Cervical circumference	0	0	0
Maxillary right first molar (16)	Overall tooth length	0	0	0
	Crown height	0	0	0
	Root length	0	0	0
	Mid mesio-distal diameter	0	0	0
	Mid buccal-palatal diameter	0	0	0
	Maximum buccal-palatal diameter	0	0	0
	Crown-to-root ratio	0.018	0.058	0.193
	Cervical circumference	0	0	0
Maxillary left central incisor (21)	Overall tooth length	0.003	0.001	0.003
	Crown height	0	0	0
	Root length	0	0	0
	Mesio-distal diameter	0.563	0.042	0
	Vestibular-palatine diameter	0	0	0
	Crown-to-root ratio	0.045	0.026	0.061
	Cervical circumference	0.013	0.004	0.003
Maxillary left lateral incisor (22)	Overall tooth length	0	0	0
	Crown height	0	0	0
	Root length	0.017	0.016	0.057
	Mesio-distal diameter	0	0	0
	Vestibular-palatine diameter	0	0	0
	Crown-to-root ratio	0.055	0.08	0.035
	Cervical circumference	0.002	0.001	0.006
Maxillary left canine (23)	Overall tooth length	0	0	0
	Crown height	0	0	0
	Root length	0	0	0
	Mesio-distal diameter	0	0	0
	Vestibular-palatine diameter	0	0	0
	Crown-to-root ratio	0.002	0.002	0.001
	Cervical circumference	0	0	0
Maxillary left first molar (26)	Overall tooth length	0	0	0
	Crown height	0.555	0.136	0.054
	Root length	0	0	0
	Mid mesio-distal diameter	0	0	0
	Mid buccal-palatal diameter	0	0	0
	Maximum buccal-palatal diameter	0	0	0
	Crown-to-root ratio	0.002	0	0.001
	Cervical circumference	0	0	0

Table 3 list the differences in morphometric variables between contralateral teeth in the DS group and in the  control group, after application of the FA2 index^3,27^. The Kruskal-Wallis test revealed differences in the crown-to-root ratios of tooth 11 versus 21, 12 versus 22, and 13 versus 23, with significantly lower values in the DS individuals than in controls. In addition, root length asymmetry of tooth 13 versus 23 was significantly smaller in the DS group than in the control group. In contrast, the differences in the crown-to-root ratio and the cervical circumference of tooth 16 versus 26 were greater in the DS group than in the controls (Table 4).

**Table 3:** Calculation of the differences in the morphometric variables between contralateral teeth in individuals with Down syndrome and controls (method: FA2 index).

Tooth	Dimension	Down Syndrome					Controls				
		Mean	Median	Standard deviation	Minimum	Maximum	Mean	Median	Standard deviation	Minimum	Maximum
Maxillary right central incisor (11) *versus* Maxillary left central incisor (21)	Overall tooth length	0.057	0.033	0.067	0	0.354	0.137	0.040	0.236	0.001	1.155
	Crown height	0.885	0.716	0.821	0	3.530	1.117	0.652	1.772	0.102	10.602
	Root length	1.246	0.866	1.345	0	5.200	0.746	0.604	0.869	0.008	4.539
	Mesio-distal diameter	1.074	0.935	1.082	0	5.314	0.923	0.713	0.681	0.098	3.223
	Vestibular-palatine diameter	0.750	0.525	0.844	0	3.736	1.256	0.962	1.748	0.067	10.337
	Crown-to-root ratio	0.380	0.245	0.441	0.018	2.331	1.636	0.730	2.244	0.073	11.184
	Cervical circumference	0.855	0.716	0.687	0	2.536	1.148	0.799	1.076	0.034	3.144
Maxillary right lateral incisor (12) *versus* Maxillary left lateral incisor (22)	Overall tooth length	1.066	0.629	1.031	0.0288	4.190	0.931	0.494	1.077	0.049	6.009
	Crown height	1.335	0.463	4.199	0.004	26.245	0.655	0.446	0.550	0.093	2.738
	Root length	1.135	0.915	0.928	0.082	3.978	0.860	0.642	0.965	0.004	4.421
	Mesio-distal diameter	1.246	0.473	2.638	0	12.180	0.746	0.468	0.839	0.052	3.307
	Vestibular-palatine diameter	1.091	0.668	1.145	0.030	4.254	0.905	0.481	1.292	0	4.630
	Crown-to-root ratio	0.532	0.387	0.464	0.011	1.873	1.479	0.787	1.638	0.103	7.455
	Cervical circumference	1.100	0.598	1.345	0.035	5.741	0.896	0.591	1.003	0.035	4.054
Maxillary right canine (13) *versus* Maxillary left canine (23)	Overall tooth length	0.825	0.633	0.603	0.084	2.507	1.179	0.774	1.152	0.016	3.979
	Crown height	0.652	0.639	0.511	0.035	1.936	1.357	0.888	1.451	0.008	4.851
	Root length	0.609	0.493	0.528	0.023	2.440	1.401	1.483	0.974	0	2.864
	Mesio-distal diameter	1.216	0.821	1.420	0	7.219	0.777	0.706	0.719	0	3.342
	Vestibular-palatine diameter	1.252	0.848	1.247	0.111	6.203	0.740	0.639	0.536	0.027	2.142
	Crown-to-root ratio	0.419	0.201	0.867	0	4.813	1.596	1.313	1.865	0.047	6.439
	Cervical circumference	0.954	0.520	1.327	0.026	7.572	1.046	0.941	1.231	0.005	5.731
Maxillary right first molar (16) *versus* Maxillary left first molar (26)	Overall tooth length	0.963	0.800	0.826	0.070	3.917	1.037	0.688	2.405	0.098	15.067
	Crown height	1.118	0.940	0.978	0.090	4.960	0.878	0.724	0.687	0.045	3.284
	Root length	1.256	1.101	1.073	0.056	4.347	0.736	0.508	0.534	0.112	2.107
	Mid mesio-distal diameter	1.048	0.961	0.913	0.040	4.195	0.950	0.900	0.667	0	3.028
	Mid buccal-palatal diameter	1.093	0.984	0.791	0.040	2.843	0.904	0.832	0.649	0	2.477
	Maximum buccal-palatal diameter	1.067	0.705	0.865	0.113	3.344	0.931	0.591	0.664	0.113	2.343
	Crown-to-root ratio	1.248	0.700	1.336	0	4.906	0.745	0.467	0.569	0	2.336
	Cervical circumference	0.884	0.754	0.755	0.011	3.372	1.119	1.333	0.779	0	2.303

**Table 4:** Statistical significance of the differences in dental morphometric variables of contralateral teeth between individuals with Down syndrome and controls (method: FA2 index).

Tooth	Dimension	T test	Wilcoxon test	Kruskal-Wallis test
Maxillary right central incisor (11) *versus* Maxillary left central incisor (21)	Overall tooth length	0.054	0.424	0.176
	Crown height	0.474	0.820	0.158
	Root length	0.060	0.072	0.158
	Mesio-distal diameter	0.473	0.937	0.614
	Vestibular-palatine diameter	0.118	0.068	0.065
	Crown-to-root ratio	0.002	0	0
	Cervical circumference	0.166	0.518	0.513
Maxillary right lateral incisor (12) *versus* Maxillary left lateral incisor (22)	Overall tooth length	0.580	0.514	0.447
	Crown height	0.329	0.899	0.922
	Root length	0.213	0.058	0.070
	Mesio-distal diameter	0.272	0.542	0.599
	Vestibular-palatine diameter	0.512	0.200	0.345
	Crown-to-root ratio	0.002	0.001	0.003
	Cervical circumference	0.458	0.816	0.797
Maxillary right canine (13) *versus* Maxillary left canine (23)	Overall tooth length	0.103	0.675	0.307
	Crown height	0.008	0.069	0.118
	Root length	0	0.001	0
	Mesio-distal diameter	0.096	0.233	0.169
	Vestibular-palatine diameter	0.024	0.115	0.173
	Crown-to-root ratio	0.001	0	0
	Cervical circumference	0.755	0.373	0.186
Maxillary right first molar (16) *versus* Maxillary left first molar (26)	Overall tooth length	0.860	0.213	0.096
	Crown height	0.223	0.351	0.402
	Root length	0.010	0.043	0.032
	Mid mesio-distal diameter	0.596	0.840	0.620
	Mid buccal-palatal diameter	0.260	0.445	0.269
	Maximum buccal-palatal diameter	0.447	0.730	0.402
	Crown-to-root ratio	0.038	0.360	0.250
	Cervical circumference	0.189	0.142	0.017

Combining the crown-to-root ratios of tooth 11 versus 21, 12 versus 22, and 13 versus 23, we developed a predictive model with an area under the curve (AUC) of 0.983 (95% confidence interval = 0.958–1) (Table 5).

**Table 5:** Discriminatory power of the generalized additive models combining various dental morphometric variables (after applying the FA2 index) to classify a specific individual as DS or non-syndromic control.

Model	Tooth	Dimension	AUC	Confidence interval
1	Maxillary right canine (13) *versus* Maxillary left canine (23)	Root length	0.8077	0.7084–0.9070
2	Maxillary right first molar (16) *versus* Maxillary left first molar (26)	Cervical circumference	0.7841	0.6811–0.8871
3	Maxillary right central incisor (11) *versus* Maxillary left central incisor (21)	Crown-to-root ratio	0.8165	0.7211–0.9118
4	Maxillary right lateral incisor (12) *versus* Maxillary left lateral incisor (22)	Crown-to-root ratio	0.7402	0.6287–0.8518
5	Maxillary right canine (13) *versus* Maxillary left canine (23)	Crown-to-root ratio	0.8519	0.7659–0.9379
6	Model 3 + Model 4 + Model 5	Crown-to-root ratio	0.9831	0.9585–1

## Discussion

In our series, the morphometric dimensions of the teeth evaluated were smaller in the DS individuals than in the controls. These results confirm the findings of other authors, who also showed that the crown dimensions of permanent teeth were smaller in individuals with DS than in healthy controls^28^, and that the roots of most anterior teeth and premolars in the DS population were shorter than in the general population^29^.

Microdontia of the permanent teeth is considered a phenotypic characteristic of DS^28^, and for comparison with non-syndromic control groups, asymmetry of dental morphometric variables in individuals with DS should not therefore be evaluated in terms of absolute right-left differences. One of the most widely used methods proposed to correct the size dependence of variability in studies of fluctuating asymmetry (FA) is the index-trait difference divided by the trait mean (FA2 index)^3^. FA2 describes fluctuating asymmetry as a proportion of trait size by estimating the between-sides variance and is hardly affected by departures from normality (skew or leptokurtosis)^17^, although it has been criticized for the apparent lack of independence between the numerator and the denominator^30^.

In the present study, we did not find greater dental crown asymmetry in DS individuals than in the controls. These results contrast with those published some decades ago by other authors, who reported greater dental asymmetry in DS^15,16^. Although those studies are of indisputable value, they carry relevant biases, such as the absence of an age- and sex-matched control group^15^ and the use of parametric tests to look for differences between DS individuals and controls^15,16^ when it is known that some dental morphometric variables do not have a normal distribution.

A novel finding of this study has been that the root length asymmetry of tooth 13 versus 23 was significantly lower in the DS group. Twin studies have shown that the canines are the teeth with greatest genetic control of dimensional variations in the general population, and they are considered the most stable teeth in the maxillary dentition^31^. Despite the biases we have indicated in those previous studies, some authors have suggested that the mesiodistal crown diameter of the maxillary right versus left canines was similar in DS individuals and in the general population^32^.

The asymmetries detected in multiroot teeth such as the maxillary first molar must be interpreted with caution, as only the dimensions of the palatine root are evaluated. It has therefore been suggested that an additional method should be devised to achieve a more accurate crown-to-root ratio of the maxillary molars^33^.

The most relevant result is that crown-to-root ratio asymmetry between the maxillary right and left central incisors, right and left lateral incisors, and right and left canines was significantly lower in the DS group. This finding is surprising for both biological and anatomical reasons, as formation of the dental crowns starts in the early weeks of intrauterine life, whereas the root portion of the tooth takes several years to develop fully and, in addition, the extra-osseous part of the tooth is particularly exposed to certain environmental aggressions, such as tooth wear. As a result, this finding requires us to revise our concepts of developmental biology such as canalization and modelization in order to analyze certain traits in individuals with DS.

The binary classification capability (DS versus control groups) of the mixed additive model that includes the crown-to-root ratio of the right versus left anterior teeth, enables us to identify individuals with DS by analyzing dental morphometric variables that can be measured easily on 2-dimensional images such as periapical or panoramic x-rays^34^. This finding could become a useful tool for the diagnosis of DS in areas such as paleopathology^35^ and paleoantropathology^36^.

The potential limitations of this study include the teeth selected, the dental variables analyzed, the method used to quantify left-right dental asymmetry and the sample size. Applying Butler’s morphogenetic field concept, the mesial tooth of each morphological tooth group is the most developmentally stable^37^; this conflicts with the results in the DS series published^13,15^, and the need to perform morphometric measurements on all teeth in the future will have to be discussed. The use of CBCT enabled us to analyze several tooth dimensions simultaneously, some of which had not previously been evaluated in DS individuals^21^. Use of the FA2 index^3^ to quantify fluctuating dental asymmetry obviates errors derived from trait size or from the use of correlations. Although it has been stated that sample sizes of several hundred are needed to detected population differences in dental asymmetry^12^, some authors have suggested a minimum sample size of 30 individuals as an empirical rule for studies of fluctuating asymmetry^38^. As the data obtained in the present study showed a low variability after applying the FA2 index, we consider that valid statistical conclusions can be drawn with the sample size used.

In summary, taking into account the limitations of this study, fluctuating dental asymmetry would appear not only not to be greater in DS individuals than in the general population, but some dental morphometric variables may also actually be more stable in individuals with trisomy 21. This offers a new perspective on the relationship between canalization, fluctuating asymmetry, and aneuploidy.

## References

[1] Waddington, C.H. *The Strategy of the Genes*: A discussion of some aspects of theoretical biology (ed. Waddington, C.H.) (George Allen and Unwin,1957).

[2] Waddington CH (1942). Canalization of development and the inheritance of acquired characters. Nature.

[3] Palmer AR, Strobeck C (1986). Fluctuating asymmetry: measurement, analysis, patterns. Annu Rev Ecol Sys.

[4] Opitz JM, Mendez HM, Hall JG (1985). Growth analysis in clinical genetics. Prog Clin Biol Res.

[5] Naugler CT, Ludman MD (1996). Fluctuating asymmetry and disorders of developmental origin. Am J Med Genet.

[6] Shapiro BL (1970). Prenatal dental anomalies in mongolism: comments on the basis and implications of variability. Ann New York Acad Sci.

[7] Blum-Hoffmann E, Rehder H, Langenbeck U (1988). Skeletal anomalies in trisomy 21 as an example of amplified developmental instability in chromosome disorders: a histological study of the feet of 21 mid-trimester fetuses with trisomy 21. Am J Med Genet.

[8] Shapiro BL (1975). Amplified developmental instability in Down’s syndrome. Ann Hum Genet.

[9] Starbuck JM, Cole TM, Reeves RH, Richtsmeier JT (2013). Trisomy 21 and facial developmental instability. Am J Phys Anthropol.

[10] Shapiro B, Gorlin R, Redman R, Bruhl H (1967). The palate and Down’s syndrome. N Engl J Med.

[11] Van Valen L (1962). A study of fluctuating asymmetry. Evolution.

[12] Smith BH, Garn SM, Cole PE (1982). Problems of sampling and inference in the study of fluctuating dental asymmetry. Am J Phys Anthropol.

[13] Garn SM, Cohen MM, Geciauskas MA (1970). Increased crown-size asymmetry in trisomy G. J Dent Res.

[14] Peretz B, Shapira J, Farbstein H, Arieli E, Smith P (1998). Modified cuspal relationships of mandibular molar teeth in children with Down’s syndrome. J Anat.

[15] Barden HS (1980). Fluctuating dental asymmetry: a measure of developmental instability in Down syndrome. Am J Phys Anthropol.

[16] Townsend GC (1983). Fluctuating dental asymmetry in Down’s syndrome. Aust Dent J.

[17] Palmer, A.R. & Strobeck, C. Fluctuating asymmetry analyses revisited. 279-319 In: (ed. Polak, M.) *Developmental Instability (DI): Causes and Consequences*. (Oxford University Press, 2003).

[18] Bell EJ, Kaidonis J, Townsend GC (2002). Tooth wear in children with Down syndrome. Aust Dent J.

[19] Banerjee A, Kamath VV, Satelur K, Rajkumar K, Sundaram L (2016). Sexual dimorphism in tooth morphometrics: An evaluation of the parameters. J Forensic Dent Sci.

[20] Palestis BG, Trivers R (2016). A longitudinal study of changes in fluctuating asymmetry with age in Jamaican youth. Symmetry.

[21] Abeleira MT (2014). Dimensions of central incisors, canines, and first molars in subjects with Down syndrome measured on cone-beam computed tomographs. Am J Orthod Dentofacial Orthop.

[22] Abeleira MT (2015). Morphometry of the hard palate in Down’s syndrome through CBCT-image analysis. Orthod Craniofac Res.

[23] Pinheiro, J. & Bates, D. *Mixed-Effects Models in S and S-PLUS. Series: Statistics and Computing* (Springer, 2000).

[24] Palmer, A. R. Fluctuating, asymmetry analyses: A primer (ed. Markow, T.A.) 335–364 In: *Developmental Instability*: *Its Origins and Evolutionary Implications* (Springer, 1994).

[25] Sherrard JF, Rossouw PE, Benson BW, Carrillo R, Buschang PH (2010). Accuracy and reliability of tooth and root lengths measured on cone-beam computed tomographs. Am J Orthod Dentofacial Orthop.

[26] Hastie, T. J. & Tibshirani, R. J. *Generalized Additive Models*. (Chapman & Hall, 1990).

[27] Palmer AR, Strobeck C (1992). Fluctuating asymmetry as a measure of developmental stability: Implications of non-normal distributions and power of statistical tests. Acta Zool Fenn.

[28] Townsend GC (1983). Tooth size in children and young adults with trisomy 21 (Down) syndrome. Arch Oral Biol.

[29] Kelsen AE, Love RM, Kieser JA, Herbison P (1999). Root canal anatomy of anterior and premolar teeth in Down’s syndrome. Int Endod J.

[30] Cuthill IC, Swaddle JP, Witter MS (1993). Fluctuating asymmetry. Nature.

[31] Osborne RH, Horowitz SL, De George FV (1958). Genetic variation in tooth dimensions: a twin study of the permanent anterior teeth. Am J Hum Genet.

[32] Barden HS (1980). Mesiodistal crown size dimensions of permanent and deciduous teeth in Down syndrome. Hum Biol.

[33] Yun HJ, Jeong JS, Pang NS, Kwon IK, Jung BY (2014). Radiographic assessment of clinical root-crown ratios of permanent teeth in a healthy Korean population. J Adv Prosthodont.

[34] Hölttä P, Nyström M, Evälahti M, Alaluusua S (2004). Root-crown ratios of permanent teeth in a healthy Finnish population assessed from panoramic radiographs. Eur J Orthod.

[35] Walker PL, Cook DC, Ward R, Braunstein E, Davee M (1991). A Down syndrome-like congenital disorder in a prehistoric California Indian. Am J Phys Anthropol.

[36] Baab KL (2016). A Critical Evaluation of the Down Syndrome Diagnosis for LB1, Type Specimen of Homo floresiensis. PLoS ONE.

[37] Butler PM (1939). Studies of the mammalian dentition – differentiation of the postcanine dentition. Proc Zool Soc London.

[38] Benítez HA, Parra LE (2011). Asimetría Fluctuante: Una herramienta morfo-funcional para medir estabilidad del desarrollo. Int J Morphol.

