# Author Correction: Reassessment of fluctuating dental asymmetry in Down syndrome

**DOI:** 10.1038/s41598-024-51672-w

**Published:** 2024-01-16

**Authors:** Marcos Matabuena Rodríguez, Pedro Diz Dios, Carmen Cadarso-Suárez, Márcio Diniz-Freitas, Mercedes Outumuro Rial, Maria Teresa Abeleira Pazos, Jacobo Limeres Posse

**Affiliations:** 1 Unit of Biostatistics, Department of Statistics and Operations Research, School of Medicine, University of Santiago de Compostela (USC), Santiago de Compostela, Spain; 2 Medical-Surgical Dentistry Research Group (OMEQUI), Health Research Institute of Santiago de Compostela (IDIS), University of Santiago de Compostela (USC), Santiago de Compostela, Spain; 3 Rede Galega INBIOEST, Santiago de Compostela, Spain

Correction to: *Scientific Reports* 10.1038/s41598-017-16798-0, published online 30 November 2017

The original version of this Article contains errors in the decimal position of data points in Table 1.

The correct Table 1 appears below.

**Table 1:** Dental morphometric variables in individuals with Down syndrome and controls (in milimeters)

Tooth	Dimension	Down syndrome					Controls				
		Mean	Median	Standard deviation	Minimum	Maximum	Mean	Median	Standard deviation	Minimum	Maximum
Maxillary right central incisor (11)	Overall tooth length	19.112	19.165	2.320	13.910	25.470	19.521	21.490	5.868	6.400	25.630
	Crown height	8.380	8.160	1.348	4.740	11.530	11.111	10.880	2.212	7.040	17.870
	Root length	10.729	10.870	1.964	5.580	14.434	11.209	11.270	2.386	6.630	14.710
	Mesio-distal diameter	7.767	7.555	0.811	5.910	9.530	8.301	8.340	0.747	7.020	9.550
	Vestibular-palatine diameter	6.765	6.685	0.663	5.080	8.920	7.412	7.370	1.070	3.580	8.790
	Crown-to-root ratio	0.461	0.454	0.093	0.248	0.844	1.223	0.996	0.709	0.541	2.662
	Cervical circumference	23.039	23.055	1.821	19.930	28.240	24.895	24.900	1.610	0.390	28.180
Maxillary right lateral incisor (12)	Overall tooth length	15.216	15.210	3.050	8.820	22.940	19.166	19.100	3.036	9.320	26.440
	Crown height	7.752	6.205	9.737	1.000	16.543	8.095	7.980	1.542	5.810	13.700
	Root length	8.960	9.045	2.734	2.280	15.250	11.382	11.710	2.705	5.320	15.910
	Mesio-distal diameter	6.510	5.805	3.237	3.270	19.290	6.567	6.520	0.856	4.620	9.300
	Vestibular-palatine diameter	5.841	5.815	0.704	4.490	7.310	6.566	6.390	0.747	4.520	7.950
	Crown-to-root ratio	0.402	0.385	0.926	0.286	0.741	0.757	0.651	0.350	0.345	1.730
	Cervical circumference	18.498	19.140	4.089	2.460	25.860	21.065	20.870	1.523	17.230	24.270
Maxillary right canine (13)	Overall tooth length	16.287	15.820	3.161	6.960	21.220	20.735	19.810	3.873	13.830	29.480
	Crown height	6.295	6.255	1.080	3.570	8.870	8.464	8.090	2.144	5.640	13.660
	Root length	9.828	10.005	2.757	2.870	15.820	12.282	11.700	3.864	5.770	20.040
	Mesio-distal diameter	6.847	6.750	0.929	4.670	10.390	7.625	7.490	0.749	6.730	9.410
	Vestibular-palatine diameter	6.754	6.850	0.600	5.190	8.670	8.006	7.840	0.556	7.060	9.350
	Crown-to-root ratio	0.433	0.391	0.182	0.258	1.396	0.771	0.633	0.419	0.353	1.811
	Cervical circumference	21.897	22.089	3.237	6.570	27.860	24.852	25.500	1.817	20.610	30.410
Maxillary right first molar (16)	Overall tooth length	18.570	18.660	1.504	14.560	22.190	21.093	20.970	1.921	11.420	23.760
	Crown height	6.007	6.080	0.571	5.080	7.440	7.070	7.260	0.645	5.110	8.250
	Root length	12.499	12.380	1.572	8.130	15.910	14.267	14.280	0.830	13.000	15.950
	Mid mesio-distal diameter	9.682	9.670	0.398	9.060	10.780	9.987	9.890	0.654	8.830	11.420
	Mid buccal-palatal diameter	9.438	9.415	0.608	8.360	10.770	10.802	10.480	1.076	8.440	13.720
	Maximum buccal-palatal diameter	9.811	9.745	0.514	8.950	10.960	11.474	11.390	0.883	9.160	13.760
	Crown-to-root ratio	0.483	0.450	0.087	0.370	0.750	0.493	0.500	0.051	0.390	0.600
	Cervical circumference	29.488	29.270	1.450	26.860	32.510	32.798	32.850	1.806	29.190	35.620
Maxillary left central incisor (21)	Overall tooth length	18.856	18.845	2.546	13.330	25.630	19.470	21.940	5.891	9.750	26.000
	Crown height	8.618	8.595	1.358	4.900	12.090	11.156	10.970	1.520	8.650	13.950
	Root length	10.105	10.150	2.427	6.020	18.660	11.243	11.420	2.404	7.980	15.000
	Mesio-distal diameter	7.893	7.810	0.839	6.470	10.800	8.328	8.110	0.624	7.400	9.860
	Vestibular-palatine diameter	6.780	6.720	0.624	5.530	8.910	7.626	7.410	0.711	6.830	9.140
	Crown-to-root ratio	0.458	0.455	0.063	0.325	0.580	1.044	0.914	0.465	0.200	2.009
	Cervical circumference	23.282	23.180	1.840	19.740	26.170	24.578	25.140	2.637	20.090	29.390
Maxillary left lateral incisor (22)	Overall tooth length	16.226	16.580	2.689	11.130	20.860	19.399	19.660	3.671	12.390	26.950
	Crown height	6.432	6.375	1.800	3.700	10.750	8.228	7.750	1.438	5.620	10.750
	Root length	9.882	9.735	2.339	5.280	14.370	11.205	12.140	3.408	5.390	18.200
	Mesio-distal diameter	5.822	5.545	1.073	4.200	9.300	6.658	6.630	1.192	4.360	8.830
	Vestibular-palatine diameter	5.879	5.665	0.797	4.700	8.260	6.682	6.640	0.914	3.820	8.260
	Crown-to-root ratio	0.391	0.392	0.794	0.240	0.613	0.788	0.665	0.302	0.371	1.708
	Cervical circumference	18.710	18.725	3.462	2.650	28.010	22.044	21.990	3.140	16.120	28.960
Maxillary left canine (23)	Overall tooth length	16.493	16.620	3.444	8.140	23.450	20.806	19.130	4.836	11.40	33.770
	Crown height	6.248	6.160	1.009	3.850	8.420	7.855	7.910	1.058	5.360	9.970
	Root length	10.150	10.075	3.039	2.460	16.350	12.935	12.020	4.284	5.660	24.550
	Mesio-distal diameter	6.557	6.645	0.591	5.120	7.940	7.682	7.480	0.690	6.180	8.950
	Vestibular-palatine diameter	6.733	6.735	0.609	5.090	8.170	8.005	8.020	0.608	6.670	9.140
	Crown-to-root ratio	0.410	0.385	0.118	0.207	0.908	0.662	0.622	0.233	0.339	1.494
	Cervical circumference	21.663	21.615	1.749	18.370	27.980	26.103	26.250	2.125	22.000	31.510
Maxillary left first molar (26)	Overall tooth length	18.751	18.585	1.539	15.010	21.680	21.380	21.070	0.982	19.890	24.110
	Crown height	6.125	6.195	0.734	4.010	7.320	7.085	6.990	0.522	6.110	8.390
	Root length	12.627	12.955	1.464	7.690	15.360	14.306	14.080	0.693	13.070	15.890
	Mid mesio-distal diameter	9.748	9.555	0.671	8.780	12.240	10.305	10.000	0.786	8.940	12.300
	Mid buccal-palatal diameter	9.598	9.635	0.664	8.000	10.900	10.585	10.510	1.048	8.620	13.950
	Maximum buccal-palatal diameter	10.015	10.015	0.517	8.730	10.890	11.556	11.460	0.962	10.170	14.370
	Crown-to-root ratio	0.486	0.460	0.109	0.310	0.950	0.491	0.490	0.037	0.410	0.560
	Cervical circumference	29.593	29.470	1.497	26.750	32.370	32.241	32.770	1.548	29.240	35.310

These errors do not affect the conclusions of the Article.

